# The Mechanism of Tigecycline Resistance in *Acinetobacter baumannii* under Sub-Minimal Inhibitory Concentrations of Tigecycline

**DOI:** 10.3390/ijms25031819

**Published:** 2024-02-02

**Authors:** Cunwei Liu, Jia Liu, Qinghui Lu, Ping Wang, Qinghua Zou

**Affiliations:** Department of Microbiology, School of Basic Medical Sciences, Peking University, Beijing 100191, China; liucunwei@pku.edu.cn (C.L.); liujia894@bjmu.edu.cn (J.L.); luqinghui@pku.edu.cn (Q.L.); wping1297@163.com (P.W.)

**Keywords:** *Acinetobacter baumannii*, tigecycline, sub-minimal inhibitory concentration (sub-MIC) antibiotics, RND efflux pump, proteome

## Abstract

The presence of sub-minimal inhibitory concentration (sub-MIC) antibiotics in our environment is widespread, and their ability to induce antibiotic resistance is inevitable. *Acinetobacter baumannii*, a pathogen known for its strong ability to acquire antibiotic resistance, has recently shown clinical resistance to the last-line antibiotic tigecycline. To unravel the complex mechanism of *A. baumannii* drug resistance, we subjected tigecycline-susceptible, -intermediate, and -mildly-resistant strains to successive increases in sub-MIC tigecycline and ultimately obtained tigecycline-resistant strains. The proteome of both key intermediate and final strains during the selection process was analyzed using nanoLC-MS/MS. Among the more than 2600 proteins detected in all strains, we found that RND efflux pump AdeABC was associated with the adaptability of *A. baumannii* to tigecycline under sub-MIC pressure. qRT-PCR analysis also revealed higher expression of AdeAB in strains that can quickly acquire tigecycline resistance compared with strains that displayed lower adaptability. To validate our findings, we added an efflux pump inhibitor, carbonyl cyanide m-chlorophenyl hydrazine (CCCP), to the medium and observed its ability to inhibit tigecycline resistance in *A. baumannii* strains with quick adaptability. This study contributes to a better understanding of the mechanisms underlying tigecycline resistance in *A. baumannii* under sub-MIC pressure.

## 1. Introduction

*Acinetobacter baumannii*, an important pathogen causing both nosocomial and community infections, is widely present in a variety of environments [1]. It also could be planted in the skin, respiratory tract, gastrointestinal tract, and genitourinary tract of patients, causing pneumonia, urinary tract infection, and bacteremia in the situation of low immunity or immunosuppression [2]. Various antibiotics were used in the treatment of *A. baumannii* infection, such as β-lactams, fluoroquinolones, tetracyclines, and aminoglycosides; however, *A. baumannii* exhibits strong environmental adaptability and could acquire resistance easily [3]. With the wide application of broad-spectrum antibiotics, the clinical separation rate of multidrug-resistant *A. baumannii* strains is increasing annually. Tigecycline, a glycylcycline class tetracycline antibiotic, has shown high in vitro antibacterial activity and is considered the last resort for the treatment of multidrug-resistant *A. baumannii* infections [4]. However, clinical isolates of *A. baumannii* with resistance to tigecycline have recently emerged [5], posing a significant challenge in clinical management [6]. Therefore, it is crucial to elucidate the complicated mechanisms underlying *A. baumannii* drug resistance [7,8].

Sub-minimal inhibitory concentration (sub-MIC) refers to a concentration of antibiotics that is lower than the minimum inhibitory concentration (MIC) but still exerts a selective effect on bacteria. Under sub-MIC conditions, low-fitness-cost bacterial mutants rather than high-fitness-cost mutants are selected by the drug [9]. Furthermore, the mutation rate in bacteria is increased under sub-MIC concentrations of certain antibiotics, leading to an accelerated evolution of drug resistance [10]. During clinical practice, drugs are excreted into the environment as unchanged forms or metabolites, resulting in the presence of trace antibiotics in the environment. In the meantime, variations in drug distribution within the body, high-dose or prolonged medication, and non-standardized medication regiments can contribute to an increased mutation rate in both pathogen and non-pathogen bacteria due to long-term exposure to sub-MIC concentrations of antibiotics. Therefore, investigating bacterial drug resistance under sub-MIC conditions has become increasingly important.

The mechanism of tigecycline resistance in *A. baumannii* was studied by several groups. For instance, Tiantuo Zhang and co-workers analyzed an efflux pump system in tigecycline-resistant *A. baumannii* [11]. K Prashanth found a strong correlation between the blaNDM-1 and armA by investigating 51 *A. baumannii* isolates [12]. However, to the best of our knowledge, no study has yet explored the mechanism of tigecycline resistance under the pressure of successive sub-MICs of tigecycline.

MS-based proteomics, the direct qualitative and quantitative studies of the complete or subset of proteins present in a species at a given condition [13], is a powerful technique extensively used in drug screening and biomarker discovery in both clinical and fundamental research [14]. The heredity and variation, drug resistance, and pathogenesis of bacteria could be greatly improved by proteomic studies.

In this study, we successfully induced 9 *A. baumannii* strains, including five tigecycline-susceptible strains, two tigecycline-intermediate strains (the MIC falls between the value of resistance and sensitivity), one tigecycline-mildly-resistant strain A54 (the MIC has just reached the level of tigecycline resistance), and one standard strain ATCC 17978 into tigecycline-resistant phenotype via incubation with successive increased sub-MICs of tigecycline. We analyzed the proteome of both key intermediate and final strains during their selection process using nanoLC-MS/MS to find out proteins associated with the tigecycline adaptability of *A. baumannii*. The findings from this study will contribute to a better understanding of the mechanisms underlying tigecycline resistance in *A. baumannii* under sub-MIC antibiotic conditions.

## 2. Results

### 2.1. Sub-MIC Selection of Tigecycline Resistant Strains

**Figure 1:**
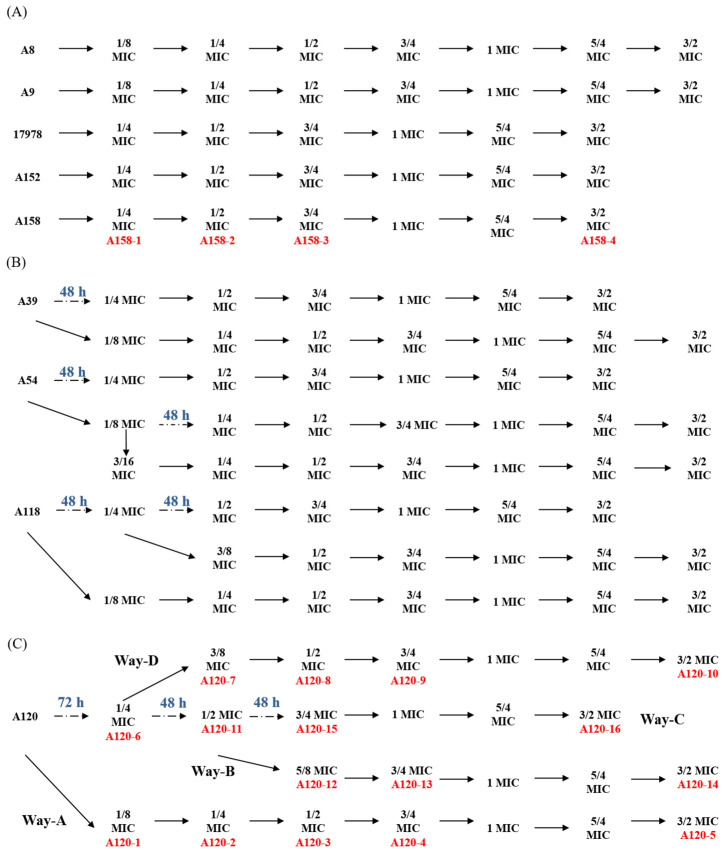
Selection processes of different clinical *A. baumannii* strains under sub-MIC tigecycline stress. The strains named such as A8, A9, etc., on the left side are the initial strains; the marks after the arrows, such as 1/8 MIC, 1/4 MIC, etc., represent the selected strains that acquired resistance to 1/8 MIC, 1/4 MIC, tigecycline, etc. The solid arrow represents a successful selection within 24 h, i.e., the strain can acquire the corresponding level of tigecycline resistance within 24 h. The dashed arrow represents a failure within 24 h, and the strain may acquire the corresponding resistance with a longer time or under a lower MIC level; see the blue number above for the corresponding selection time. The red numbers A158-1 and A120-1 in the selection processes of A158 and A120 indicate the strain subjected to proteomic analysis. The initial strain A120 is A120S, and the strains of the four selection processes, namely A120-10, A120-16, A120-14, and A120-5, belong to A120R. The same goes for A158. (**A**) Strains can acquire a higher tigecycline resistance within 24 h. (**B**) Strains can acquire a higher tigecycline resistance within 48 h or at a lower MIC. (**C**) Strains can acquire a higher tigecycline resistance within 72 h or at a lower MIC.

We chose five tigecycline-susceptible strains (A118, A120, A39, A152, and A158, with MIC to tigecycline ≤2 ng/μL), two tigecycline-intermediate strains (A8 and A9, with MIC to tigecycline 2~8 ng/μL), one tigecycline-mildly-resistant strain A54 (with MIC to tigecycline = 8 ng/μL) and one reference strain ATCC 17978 to investigate tigecycline resistance in different clinical A. baumannii isolates under sub-MIC stress systematically. Specific selection processes and results are illustrated in Figure 1. To our surprise, there are significant differences in the selection results among these clinical *A. baumannii* isolates. ATCC 17978, A152, and A158 had quick adaptabilities to sub-MIC tigecycline, as they could acquire corresponding tigecycline resistance within 24 h as the concentration of tigecycline increased by every 1/4 MIC. A8 and A9 could not acquire tigecycline resistance even when the time was prolonged to 72 h when the initial tigecycline concentration was 1/4 MIC. However, when the concentration was reduced to 1/8 MIC, A8 and A9 could acquire resistance within 24 h and could acquire tigecycline resistance at the following time points. A39, A54, and A118 could acquire resistance within 48 h with 1/4 MIC or within 24 h with 1/8 MIC tigecycline, and tigecycline-resistant phenotypes could be successfully selected in the following process. The acquisition of tigecycline-resistance of A120S to A120R was the hardest among these nine strains. It took 72 rather than 24 h for A120 to acquire resistance in 1/4 MIC, and more time was also needed when the tigecycline concentration increased to 1/2 and 3/4 MIC; see Figure 1C for details. Overall, there is a great difference among different strains.

### 2.2. Proteomic Analysis

**Figure 2:**
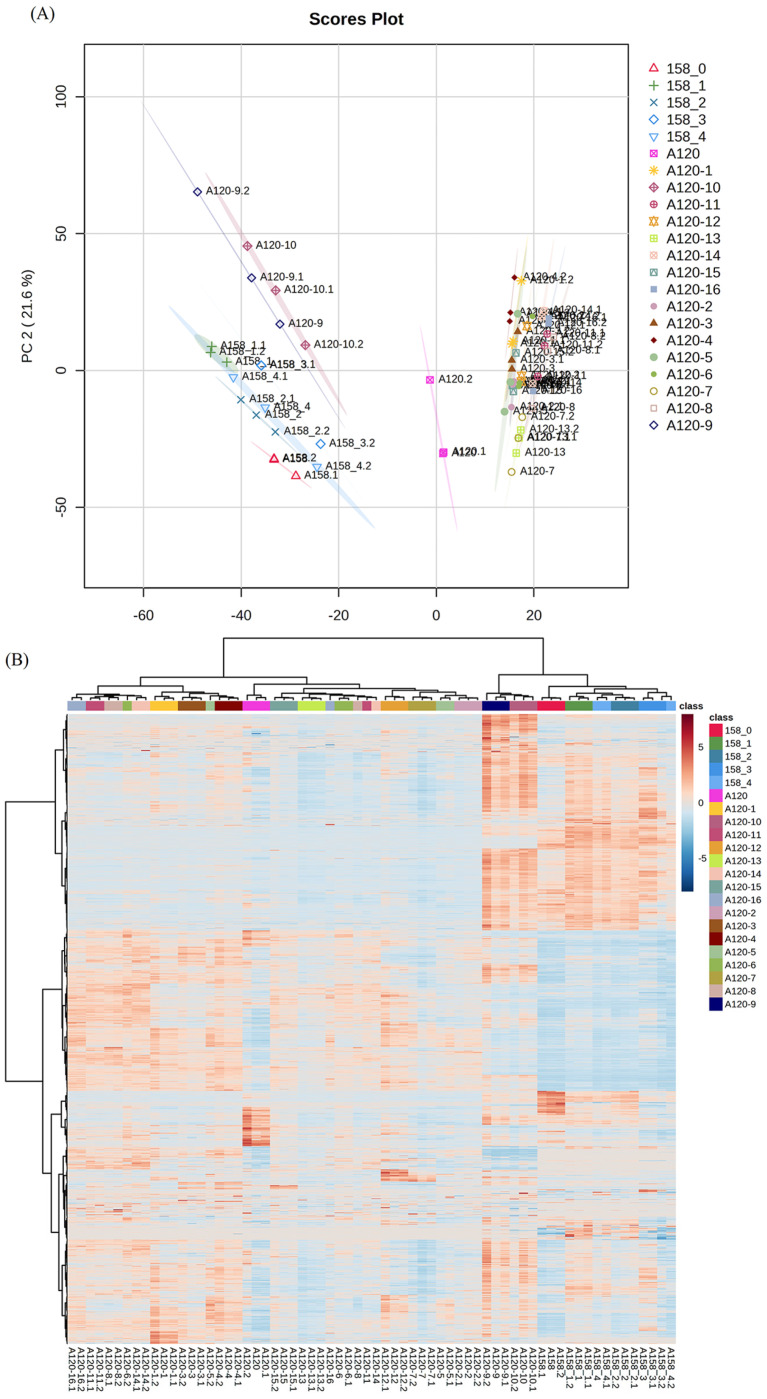
(**A**) PCA score plot of the proteome from the key intermediate and final strains in the A158 and A120 selection processes. (**B**) Clustering heat map by Euclidean correlation of the samples.

To further understand the mechanism underlying the different adaptabilities of various *A. baumannii* strains under sub-MIC of tigecycline, we chose two strains with the greatest difference in tigecycline adaptability, A158 and A120, for proteomic analysis. The key intermediate and final strains during their selection process, i.e., the strains numbered A158-1 and A120-1 in Figure 1, were analyzed using nanoLC-MS/MS (*n* = 3 for each strain). In general, more than 2600 proteins were detected in all strains. Figure 2A shows the principal component analysis (PCA) score plot of all strains analyzed. As expected, each strain was grouped tightly. All key intermediate and final strains in the A158 selection process were located on the left side, while the key intermediate and final strains in the A120 selection process were on the right except A120-9 and A120-10, which were situated close to the A158 group. Figure 2B showcases the clustering heatmap from these strains and displays the relationship among strains more clearly. Again, A120-9 and A120-10 were closer to the A158 strains than to the other A120 strains. The final strains A120-5, A120-10, A120-14, and A120-16 were not clustered together as expected.

**Figure 3:**
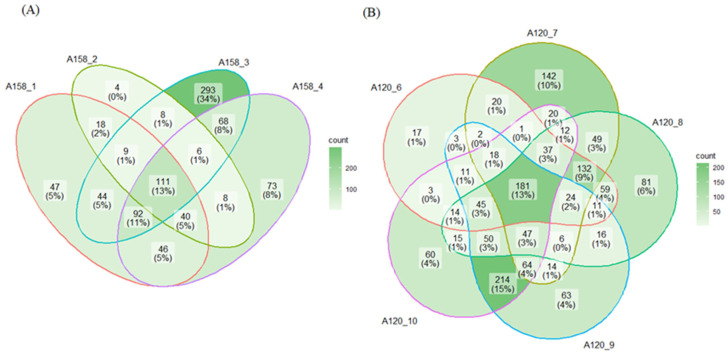
Venn diagrams represent proteins identified in (**A**) the A158 selection process and (**B**) the A120 selection process way D.

To recognize the mechanism of tigecycline resistance in the A158 selection process, we screened and obtained the proteins that significantly changed in the process strains and final strain compared with the original strain, viz. A 158-0. A four-way Venn diagram revealed 111 commonly significantly changed proteins expressed in the A158 selection process; see Figure 3A. Interestingly, the strain with the most differential proteins was A158-3, with 293 proteins found exclusively in it, which corresponded to 3/4 MIC, rather than the final strain with 3/2 MIC. This phenomenon pointed out that 3/4 MIC may be the critical node in the A158 selection process. Similarly, we screened and obtained the proteins that significantly changed in the process strains and final strain compared with the original strain in each way in the A120 selection process. In analogy to before, A120-9 and A120-10 may be the critical nodes in the A120 selection process in way D, demonstrated in Figure 3B. In A, B, and C, the intersection with the most differential proteins was in the center, the common area of all process strains and the final strain.

**Figure 4:**
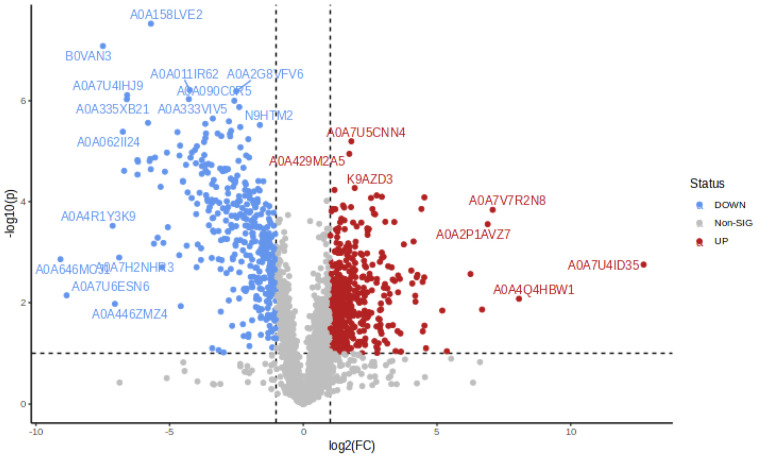
Volcano plot representing proteins identified in the A158-3 and the original strains.

As the A158-3 was the critical node in the A158 selection process, we constructed a volcano plot of A158-3 and the original strain to pinpoint the disturbed proteins, see Figure 4. The red dots represent up-regulated proteins in A158-3 compared with A158-0, while the blue dots represent down-regulated proteins. Protein with UniProt accession A0A2P1AVZ7 was one of the most up-regulated proteins in Figure 4, which was characterized as a multidrug efflux RND transporter periplasmic adaptor subunit AdeA. Prompted by this finding, we calculated the expression of AdeA and AdeB in all process strains and final strains, and the results are listed in Table 1. In the A158 selection process, AdeA was increased from node 1/4 MIC and reached a peak value of 3/4 MIC. In addition, AdeB was also increased on the node of 3/4 MIC. In contrast, AdeA was not observed to be increased continuously in the A120 selection process and only up-regulated in strains A120-9 and A120-10, which was in line with Figure 3B. AdeB was the mainly elevated protein in the A120 selection process; however, it was only up-regulated on the node near the final strain of each selection way; see Table 1 for details.

The horizontal dashed line represents the level of significance (*p*-value 0.05), and the vertical dashed lines represent 0.5 and 2-fold change.

### 2.3. PCR and qRT-PCR of adeABC in Different Strains

**Figure 5:**
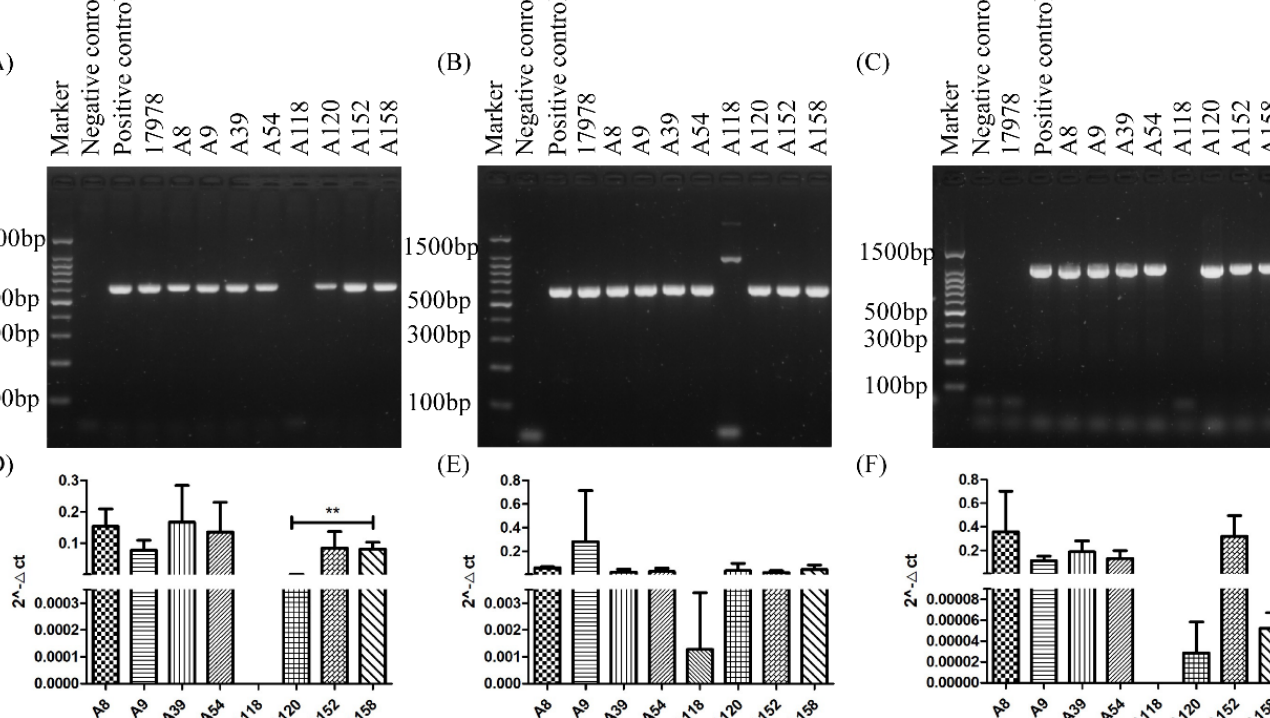
PCR and qRT-PCR of *adeABC* in clinical strains. PCR results of *adeA* (**A**), *adeB* (**B**), and *adeC* (**C**). qRT-PCR results of *adeA* (**D**), *adeB* (**E**), and *adeC* (**F**). ** *p* < 0.001.

To test our hypotheses, we designed an *adeABC* primer to detect these three genes in the original stains. Figure 5A–C shows the PCR and quantitative real-time PCR (qRT-PCR) data from *adeABC* in clinical *A. baumannii*. All original strains contained *adeABC* except ATCC 17978 (without *adeC*) and A118 (without *adeA* and *adeC*). We also performed qRT-PCR of *adeABC* in these clinical strains, shown in Figure 5D–F. There were no significant differences in the expression of *adeB* and *adeC* between A158 and A120; however, the expression of *adeA* was dramatically decreased in A120 compared with A158.

We further performed qRT-PCR on A39 and A152, which had the greatest difference in tigecycline adaptability among these strains, second to A158 and A120. If the AdeABC efflux pump system were involved in tigecycline resistance, their expressions were supposed to be higher in A152, which was easier to select for tigecycline-resistant phenotypes than A39. The results are displayed in Figure S1 in Supplementary Material. As expected, although the expression of adeB was similar between A39 and A152, both adeA and adeC were up-expressed in A152 compared with A39.

### 2.4. Inhibition of Efflux Pump

To verify the proteomic result that the AdeABC efflux pump system is critical in the acquisition of tigecycline-resistance in *A. baumannii*, we compared the MICs detected in A120, A158, and the key strains in the selection process with and without CCCP, an efflux pump inhibitor, and the results are listed in Table 2. For A120 series strains, after incubating with CCCP, only the MICs of A120-9, A120-14, and A120-16 were reduced by half. In contrast, the MICs of the other strains remained unchanged, reflecting little significant influence of CCCP on tigecycline resistance. However, for A158 series strains, the MICs of all strains, including both the original and key strains in the selection process, decreased to 50% after incubating with CCCP.

After that, we repeated the tigecycline selection process of A158 and A120 but added both tigecycline and CCCP and determined the OD570 after 24 h culture to figure out the effect of CCCP on tigecycline resistance in *A. baumannii*. As shown in Figure S2A, CCCP could suppress the selection of A158 in the early stage until 3/4 MIC. In addition, on the node of 1 MIC and 3/2 MIC, the selection of A158 was also suppressed significantly by CCCP. However, for A120 series strains, CCCP could not suppress the selection of A120 on multiple nodes in various ways.

### 2.5. Growth Curve and Biofilm

**Figure 6:**
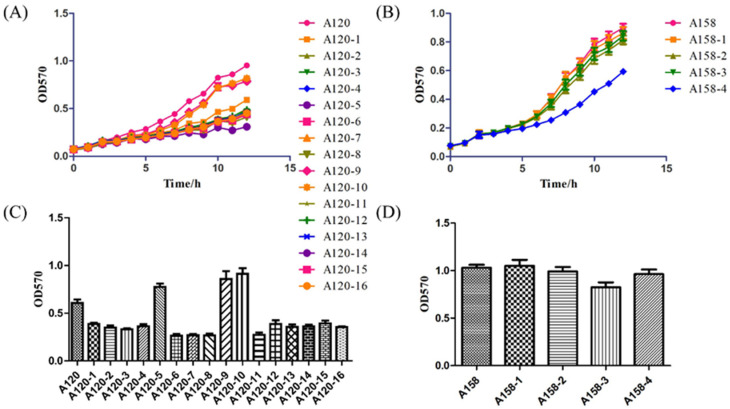
Growth curve determination and biofilm formation. Growth curve results of (**A**) A120 and (**B**) A158 in LB medium. Biofilm formation of (**C**) A120 and (**D**) A158.

Provided the AdeABC efflux pump system was not the only reason for tigecycline resistance in *A. baumannii*, we determined the growth curve and biofilm of both A120 and A158 series strains. Growth curves were performed in both LB and M9 medium. There was no significant difference in the growth in the M9 medium; however, these stains exhibited quite different growth curves in the LB medium, see Figure 6A,B. For A120 series strains, all process strains grew slowly compared with the original strain except A120-9 and A120-10, which had the same growth ability as the original strain. For A158 series strains, the strains in the early stages of the selection process displayed similar growth curves. In contrast, the strain induced by 3/2 MIC exhibited significant growth retardation from the fifth hour.

When it comes to biofilm formation, see Figure 6C,D, there was no significant difference for the A158 series strains. For A120 series strains, as tigecycline concentration increased, the ability of biofilm formation decreased first and then increased in the selection processes A, B, and C. In addition, the biofilm formation abilities of the final strains in the selection process A and B were much stronger than those of the original strain. The process strains in the selection process D showed lesser biofilm formation abilities compared with the original strain, which may partially account for the greater difficulty in this selection process.

## 3. Discussion

In clinical practice, the exposure of bacteria to sub-MIC antibiotic environments is inevitable due to inappropriate medications, such as long-term or high-dose use of antibiotics, leading to the development of the resistance of bacteria. Based on this phenomenon, we simulated the sub-MIC tigecycline environment in our lab and induced tigecycline-susceptible and tigecycline-intermediate strains to tigecycline-resistant phenotypes by successive increased sub-MICs of tigecycline. Our results indicated that the ability to develop resistance to tigecycline under sub-MIC conditions varies among different strains. Surprisingly, the tigecycline-mildly-resistant strain A54 did not exhibit a higher propensity to acquire tigecycline resistance. Therefore, there appears to be no direct correlation between the initial susceptibility to tigecycline and the ability to acquire higher levels of tigecycline resistance. The factors influencing the acquisition of tigecycline resistance under sub-MIC conditions warrant further investigation.

PCA analysis of the proteomic results indicates a similar proteome in the initial, intermediate, and final strains. The final strains A120-5, A120-10, A120-14, and A120-16 were not clustered together as expected, which reflected complexity in the mechanism of tigecycline resistance in *A. baumannii*. Upon further analysis, we found that AdeA and AdeB were differentially expressed during the selection process in A120 and A158. These results indicated that the AdeABC efflux pump system was probably related to the propensity of acquired resistance to tigecycline under sub-MIC tigecycline conditions. Our study is in line with previous studies in which AdeB was reported to be positively related to the tigecycline and cefotaxime resistance in *A. baumannii* [15]. This could be explained by the structure of AdeABC [16]: AdeB is the main functional part of the efflux pump system, while AdeA, as an outer membrane protein, helps AdeB with drug efflux. It was suggested that the propensity of acquired tigecycline resistance was associated with the integrity of the AdeABC efflux pump system. A118 is a strain that makes it hard to acquire tigecycline resistance. We found that adeA and adeC were absent in this strain. Therefore, it can be speculated that with the lack of AdeA and AdeC proteins, A118 could not construct a complete efflux pump structure, leading to an impaired drug efflux function and a decreased adaptability to tigecycline. It was congruous with the difficulty in the A118 selection process. qRT-PCR showed that the expression of adeA was dramatically decreased in A120 compared with A158, which may be basically why A120 was much more difficult to select for tigecycline-resistant phenotypes than A158. All this work supports the assumption that the main drug resistance mechanism in *A. baumannii* is the efflux pump system under sub-MIC pressures. When incubated with the efflux pump inhibitor CCCP, the MICs of A120 decreased much less than A158, indicating that the complicated mechanism involved and the AdeABC efflux pump system played a more important role in strains that can acquire a quick resistance rather than strains that can acquire a slow resistance under the sub-MIC condition.

**Figure 7:**
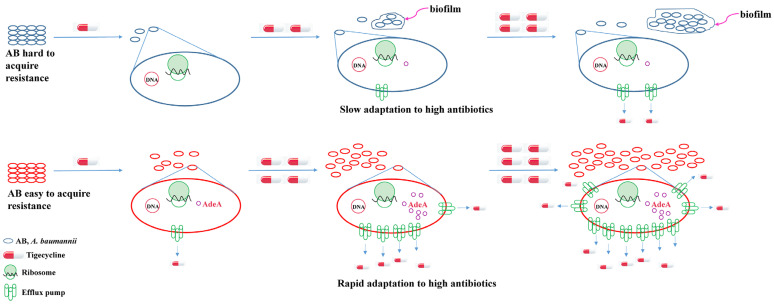
Schematic summary of tigecycline resistance mechanism under sub-MIC pressure. The upper portion stands for *A. baumannii,* which is hard to acquire resistance, and the main tigecycline resistance mechanism is biofilm formation. The lower portion stands for *A. baumannii,* which easily acquires resistance. Strains that can express high levels of AdeA and AdeB can export tigecycline out of the cells more efficiently and thus can acquire tigecycline resistance in a short time.

The impact of sub-minimal inhibitory concentration antibiotics on antibiotic resistance is a topic of great concern. Studies have already revealed the importance of efflux pumps in antibiotic resistance in bacteria. However, there is still a certain knowledge gap regarding the impact of sub-minimal concentration antibiotics on bacterial resistance, especially the role of efflux pumps. This study indicated that efflux pumps remain an important way for bacteria to acquire resistance under sub-minimal concentration antibiotics. The mechanism of tigecycline resistance under sub-MIC conditions was summarized as shown in Figure 7. Although previous studies have shown that efflux pump systems play a role in tigecycline resistance *in A. baumannii* [17,18], to our knowledge, this study is the first to reveal the tigecycline resistance mechanism under sub-MIC pressure. Our results provide detailed insights into the changes in efflux pump activity under sub-minimal inhibitory concentrations of antibiotics in different strains, further confirming the role of efflux pumps in tetracycline resistance.

## 4. Materials and Methods

### 4.1. Bacteria Strains and Antibiotic Susceptibility Test

Five clinically isolated tigecycline-susceptible strains (A118, A120, A39, A152, and A158), two tigecycline-intermediate strains (A8 and A9), one tigecycline-mildly-resistant strain A54 and one standard strain ATCC 17978 were included in this study. The susceptibility of bacteria to tigecycline was tested using the broth dilution method.

### 4.2. Selection of Tigecycline-Resista17978nt Phenotype with Sub-MIC Tigecycline

The tigecycline-susceptible, intermediate, and mildly resistant strains were cultured overnight and transferred with a ratio of 1:100 to fresh MH broth containing successive increasing tigecycline to select strains with tigecycline-resistant phenotypes. Briefly, a single colony of the initial strains was picked up and first cultured with 1/4 MIC tigecycline in tubes for 24 h; if there was bacterial growth in the culture, then it was transferred to fresh MH broth containing a higher concentration tigecycline with 1/2 MIC tigecycline for 24 h, if there are bacteria growth in the culture, it was transferred to 3/4 MIC, 1 MIC, and 3/2 MIC of tigecycline accordingly every 24 h. If growth cessation was observed at some time point, the concentration of tigecycline was reduced to one-half for cultivation or cultivated for a longer time within 72 h. When growth arrived at 3/2 MIC, the MIC of the bacteria was tested. If the MIC ≥ 8 μg/mL, the strain was considered resistant to tigecycline.

### 4.3. Experimental Design and Statistical Rationale

We chose 20 strains, including the key intermediate and final strains, during both the A158 and A120 selection processes for proteomic analysis. Two original strains were used for controls. Due to the more controllable growth conditions of bacteria, their proteome is relatively stable compared with humans and animals. In our study, three biological replicates were analyzed for proteomics for each strain.

Several valuable tools in proteomic analysis, such as PCA analysis, heatmaps, volcano plots, and Venn diagrams, were used for data treatment. They provide justifications by facilitating the understanding of protein expression patterns, discovering differentially expressed proteins, and identifying relationships between samples. These tools aid researchers in gaining deeper insights and interpretations of proteomic data.

### 4.4. Proteomic Analysis

After extracting and purifying the protein of the strains, the proteomics analysis was carried out by EASY nLC coupled with a Q Exactive Plus mass spectrometer. Data were collected in a data-dependent top 20 scan mode. Survey full-scan MS spectra (mass range *m*/*z* 300 to 1800) were acquired with resolution R = 70,000 and AGC target 3 × 10^6^. MS/MS fragmentation was performed using high-energy c-trap dissociation (HCD) with resolution R = 17,500 and AGC target 2 × 10^5^. The parent ions were fragmented with an NCE of 28.

Then, these raw data were analyzed using Proteome Discoverer version 2.4.0.305 (Thermo Fisher Scientific, Waltham, MA, USA), where the mass spectral files were queried using the Sequest HT search engine against the UniProt Acinetobacter + baumannii.fasta (354302 SEQUENCES, 25 June 2021). The mass tolerance of precursor ions was set as 10 ppm, and the mass tolerance of MS/MS was set as 0.02 Da. The false discovery rates for protein and peptides were set at a maximum of 1%. Trypsin was used to generate peptides. Two missed and/or non-specific cleavages were permitted. Dynamic Modification: Oxidation/+15.995 Da (M); N-Terminal Modification: Acetyl/+42.011 Da (N-Terminus); N-Terminal Modification: Met-loss/−131.040 Da (M); N-Terminal Modification: Met-loss + Acetyl/−89.030 Da (M). Static Modification: Carbamidomethyl/+57.021 Da (C).

### 4.5. qRT-PCR

RNAprep Pure bacterial total RNA extraction kit (TIANGEN, Beijing, China) was used to extract the RNA of the strains. After reverse transcription, it is equipped with a 20 μL reaction system with 1~10 ng cDNA as the template. A standard two-step procedure was used for amplification.

### 4.6. Efflux Pump Inhibition Experiment

The efflux pump inhibitor CCCP (TargetMol, Shanghai, China) was used for the efflux pump inhibition experiment. The MICs of the bacteria were tested by the dilution method in a cation-regulated MH liquid medium containing 10 mg/mL CCCP. The efflux pump inhibitory phenotype was considered positive when the MIC of a certain strain decreased by more than or equal to two folds in the presence of an efflux pump inhibitor.

### 4.7. Growth Curve Measurement Experiment

A single colony of the strain was selected and cultured overnight in a Luria-Bertani (LB) liquid medium. The OD570 was measured with a spectrophotometer. Three sets of biological replicates were conducted. The LB culture media without bacteria was used as a blank control.

### 4.8. Biofilm Formation Assay

Biofilm quantifications were performed after 12 h growth in LB medium using the crystal violet method. Briefly, tubes were stained with 0.1% crystal violet for 20 min and rinsed with ultrapure water. Then, biofilms were dissolved in 95% ethanol after drying. The optical density at 570 nm (OD570) was measured. Experiments were performed three times with similar results.

## 5. Conclusions

In summary, in this study, we successfully selected nine *A. baumannii* strains into tigecycline-resistant phenotype via incubation with successive sub-MICs of tigecycline. The proteome of two strains with the greatest difference in tigecycline adaptability, A158 and A120, were detected and analyzed. It was found that RND efflux pump AdeABC was associated with the tigecycline adaptability of *A. baumannii* under sub-MIC conditions, which was confirmed by the PCR and qRT-PCR results and efflux pump inhibition experiments using an efflux pump inhibitor CCCP. Strains that can express high levels of AdeA and AdeB can acquire tigecycline resistance in a short time; otherwise, the opposite is true. In addition, this biofilm also accounts for tigecycline resistance, especially in the A120 strains, indicating the complexity of the tigecycline-resistant mechanism in *A. baumannii*.

## Figures and Tables

**Table 1:** Expression of AdeAB in strains in the selection process.

Strain	AdeA (Abundance Ratio)	AdeB (Abundance Ratio)	Strain	AdeA (Abundance Ratio)	AdeB (Abundance Ratio)
A158-1	48.209	-	A120-7	-	-
A158-2	24.205	-	A120-8	-	-
A158-3	99.772	18.84	A120-9	100	14.195
A158-4	27.582	-	A120-10	100	-
A120-1	-	-	A120-11	-	15.276
A120-2	-	-	A120-12	-	26.619
A120-3	-	-	A120-13	-	0.107
A120-4	-	21.67	A120-14	-	20.785
A120-5	-	27.919	A120-15	-	0.113
A120-6	-	-	A120-16	-	41.994

- standards for no significant difference in protein expression.

**Table 2:** The impact of efflux pump inhibitor on strain MICs.

Strain	Tigecycline MIC (ng/μL)	Tigecycline + CCCP MIC (ng/μL)	Strain	Tigecycline MIC (ng/μL)	Tigecycline + CCCP MIC (ng/μL)
ATCC 25922	0.25	-	A120-11	8	8
A120	0.5	0.5	A120-12	8	8
A120-1	2	2	A120-13	8	8
A120-2	4	2	A120-14	16	8
A120-3	8	8	A120-15	8	8
A120-4	16	16	A120-16	16	8
A120-5	16	16	A158	0.5	0.25
A120-6	1	1	A158-1	16	8
A120-7	4	4	A158-2	16	8
A120-8	4	4	A158-3	32	16
A120-9	64	32	A158-4	128	64
A120-10	256	256			

## Data Availability

All data were available when requested.

## Supplementary Materials

The supporting information can be downloaded at: https://www.mdpi.com/article/10.3390/ijms25031819/s1.
