# Supplementary material for: The Mechanism of Tigecycline Resistance in Acinetobacter baumannii under Sub-Minimal Inhibitory Concentrations of Tigecycline
Source: Int J Mol Sci. 2024 Feb 2;25(3):1819. doi: 10.3390/ijms25031819 (PMC10855123; doi:10.3390/ijms25031819)
Supplement: Supplementary file 1 [file ijms-25-01819-s001.zip › ijms-2788751-supplementary.pdf]

Proteomics-based study on the mechanism of tigecycline resistance in *Acinetobacter baumannii*

Cunwei Liu†, Jia Liu†, Qinghui Lu, Ping Wang, Qinghua Zou*

Department of Microbiology and Infectious Disease Center, School of Basic Medical Sciences, Peking University Health Science Center, Beijing, 100191, China

*** Corresponding author contact information**

Qinghua Zou, Ph.D.

Department of Microbiology and Infectious Disease Center, School of Basic Medical Sciences, Peking University Health Science Center

Address: 38 Xueyuan Road, Haidian District, Beijing 100191, China

Phone: 86-10-82805070

E-mail: zouqinghua@bjmu.edu.cn

† Cunwei Liu and Jia Liu contributed equally to this work.

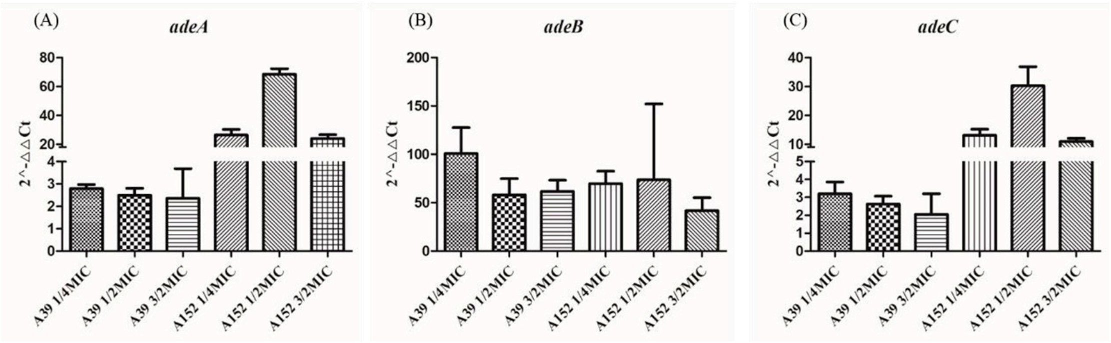

Figure S1. qRT-PCR results of *adeA* (A), *adeB* (B) and *adeC* (C) in A139 and A152 strains.

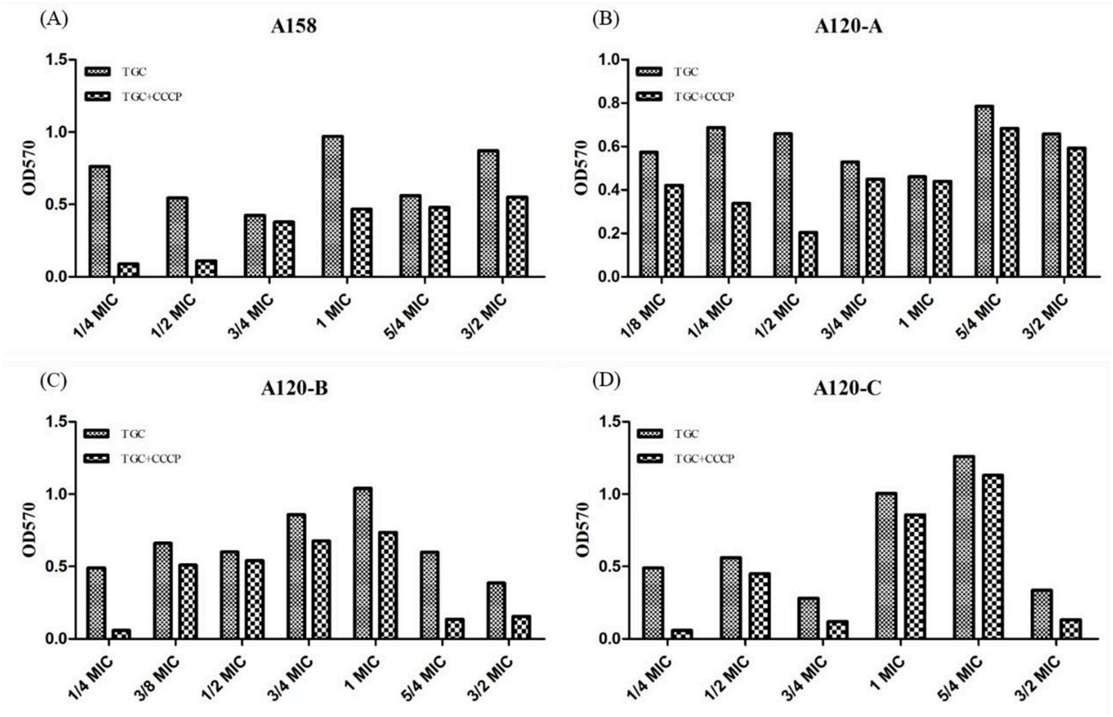

Figure S2. OD_{570} of bacteria in tigecycline induction process with only tigecycline and tigecycline + CCCP. TGC, tigecycline; CCCP, carbonyl cyanide m-chlorophenyl hydrazine.
